# Technological Advances and Market Developments of Solid-State Batteries: A Review

**DOI:** 10.3390/ma17010239

**Published:** 2024-01-01

**Authors:** Felix Thomas, Lauren Mahdi, Julien Lemaire, Diogo M. F. Santos

**Affiliations:** 1 Mechanical Engineering Department, Instituto Superior Técnico, Universidade de Lisboa, 1049-001 Lisbon, Portugal; felix.thomas@tecnico.ulisboa.pt (F.T.); lauren.mahdi@tecnico.ulisboa.pt (L.M.); julien.lemaire@tecnico.ulisboa.pt (J.L.); 2 Center of Physics and Engineering of Advanced Materials, Laboratory for Physics of Materials and Emerging Technologies, Chemical Engineering Department, Instituto Superior Técnico, Universidade de Lisboa, 1049-001 Lisbon, Portugal

**Keywords:** solid-state batteries, solid-state electrolytes, lithium-ion batteries, market outlook, production economics

## Abstract

Batteries are essential in modern society as they can power a wide range of devices, from small household appliances to large-scale energy storage systems. Safety concerns with traditional lithium-ion batteries prompted the emergence of new battery technologies, among them solid-state batteries (SSBs), offering enhanced safety, energy density, and lifespan. This paper reviews current state-of-the-art SSB electrolyte and electrode materials, as well as global SSB market trends and key industry players. Solid-state electrolytes used in SSBs include inorganic solid electrolytes, organic solid polymer electrolytes, and solid composite electrolytes. Inorganic options like lithium aluminum titanium phosphate excel in ionic conductivity and thermal stability but exhibit mechanical fragility. Organic alternatives such as polyethylene oxide and polyvinylidene fluoride offer flexibility but possess lower ionic conductivity. Solid composite electrolytes combine the advantages of inorganic and organic materials, enhancing mechanical strength and ionic conductivity. While significant advances have been made for composite electrolytes, challenges remain for synthesis intricacies and material stability. Nuanced selection of these electrolytes is crucial for advancing resilient and high-performance SSBs. Furthermore, while global SSB production capacity is currently below 2 GWh, it is projected to grow with a >118% compound annual growth rate by 2035, when the potential SSB market size will likely exceed 42 billion euros.

## 1. Introduction

Batteries are becoming increasingly essential in modern society to power many devices, including smartphones, laptops, electric cars, and renewable energy grids. As the demand for portable electronics and electric vehicles (EVs) continues to rise, the need for high-performing, long-lasting, and safe batteries is becoming more pressing. Advances in battery technology can significantly impact how we live and work, from enabling sustainable energy to reducing our reliance on fossil fuels. When John B. Goodenough and his team published the famous paper “A new cathode material for batteries of high energy density” in 1980 [1], they could not have foreseen the far-reaching consequences of their work. Since then, lithium-ion batteries (LIBs) have established themselves as the leading technology in the global battery market due to their superior energy density, extended cycle life, and low self-discharge rates. They are employed in various applications, such as smartphones, laptops, electric vehicles, and renewable energy storage systems [2]. Furthermore, they have experienced a significant decline in cost, with Bloomberg NEF’s 2021 battery price survey reporting an 89% reduction in prices since 2010 and an increase in installed capacity values (with a global installed capacity of over 800 GWh as of 2020), which highlights the rapid progress of battery technology in recent years [3,4].

Unfortunately, LIBs suffer from safety concerns related to their potential for thermal runaway and fire, especially when overcharged or exposed to high temperatures, as well as relatively long charging times [5]. Therefore, intensified research in battery technologies is inevitable. Among upcoming and highly promising battery technologies is the so-called solid-state battery (SSB), a novel battery technology that is vital in shaping the future of energy and sustainability. By using solid electrolytes instead of liquid ones, SSBs differ significantly from LIBs due to their enhanced safety, higher energy density, and longer lifespan [6,7,8,9,10]. These unique attributes make SSBs appealing for applications with specific requirements.

One such area is the transportation industry, encompassing EVs and aerospace. EVs, a prominent sector, stand to benefit significantly from SSBs, driving substantial investment and research in this direction. Although LIBs currently dominate electric battery vehicles, SSBs offer distinct advantages, notably fast charging and improved safety. Solid electrolytes eliminate the risk of electrolyte leakage or vaporization and mitigate the potential for flammable organic solvents. They also prevent side reactions between electrodes and electrolytes that could lead to dendrite formation. Moreover, the higher energy density of SSBs can extend the range of electric vehicles, enhancing their viability for long-distance travel. Major companies like Toyota, Honda, Nissan, Ford, BMW, and Volkswagen have actively pursued SSB development for electric vehicles.

SSBs have already found utility in aerospace applications due to their lighter weight, compactness, and higher energy density. These attributes make them suitable for energy storage in spacecraft. The safety features of SSBs make them particularly appealing for this application, in contrast to conventional LIBs, which are lighter and more compact but often have lower safety levels. Solid electrolytes enable SSBs to withstand extreme temperatures in space environments. Certain SSBs, such as lithium-air batteries, can function at temperatures as low as −73 °C [11], while others, like lithium-oxygen batteries, can operate at temperatures up to 120 °C [12]. In addition to the transport sector, there is a growing demand for batteries offering the advantages provided by SSBs in various industries, such as medical devices and consumer electronics. These sectors find SSBs to be compelling choices for their specific needs. The multiple applications discussed underscore the potential of SSBs and their significance for the future.

This review provides an overview of SSB technology, primarily focusing on the status of electrolyte and electrode material research and market perspectives. First, the currently most relevant materials employed are presented. Subsequently, the global battery market, specifically focusing on emerging SSB technologies, is introduced. The current and projected SSB market size, its economics, as well as an overview of the key players and collaborators are shown.

## 2. Solid-State Electrolyte Materials

**Figure 1:**
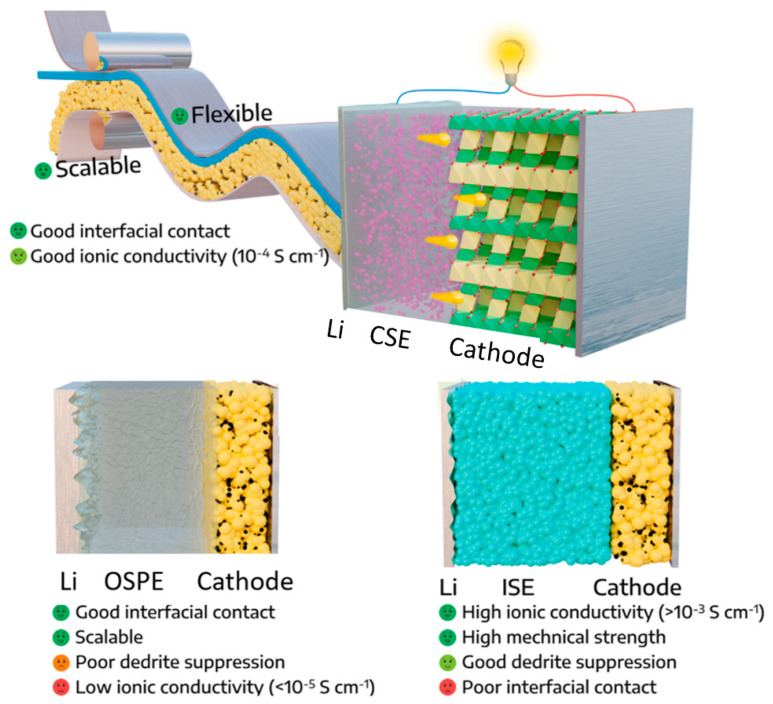
Comparison of the structure and properties of inorganic solid electrolytes (ISEs), organic solid polymer electrolytes (OSPEs), and composite solid electrolytes (CSEs) [15]. Reprinted with permission from Elsevier.

SSBs are an emerging technology that has the potential to revolutionize the energy storage industry. Unlike traditional LIBs, which use a liquid electrolyte to transport ions between the cathode and anode, SSBs use a solid-state electrolyte (SSE) to perform the same transport function. As shown in Figure 1, SSEs used in rechargeable batteries can be divided into three categories based on chemical composition: inorganic solid ceramic electrolytes, organic solid polymer electrolytes, and solid composite electrolytes, a combination of the first two material classes [7,13,14].

Inorganic solid electrolytes (ISEs) are typically made from lithium ceramics such as lithium aluminum titanium phosphate (LATP). They offer high ion conductivity and thermal stability but can be brittle and difficult to manufacture [7].

Organic solid polymer electrolytes (OSPEs) are made from polymers such as polyethylene oxide (PEO) or polyvinylidene fluoride (PVDF). They offer good mechanical flexibility and processability, but lower ion conductivity than inorganic solid ceramic electrolytes [8].

Composite solid electrolytes (CSEs) combine inorganic ceramic materials with organic polymers to achieve high ion conductivity and good mechanical properties. They can be designed to have specific properties by varying the composition and structure of the materials.

There are critical factors for the success of SSEs in SSBs. In essence, optimal SSEs should exhibit characteristics such as extremely low electronic conductivity (<10^−10^ S cm^−1^) coupled with high Li^+^ conductivity (>10^−3^ S cm^−1^) [16]. Furthermore, they should demonstrate favorable chemical compatibility with electrodes, a broad electrochemical stability range, and exceptional thermal stability [17]. Researchers are working on various strategies to improve Li^+^ conductivity, such as optimizing the microstructure of the materials, incorporating dopants, and using hybrid materials [11,12,17,18].

### 2.1. Inorganic Solid Electrolytes

**Figure 2:**
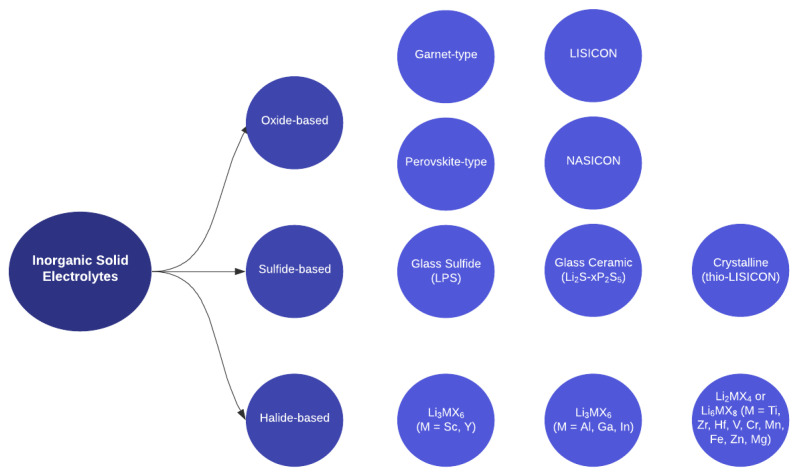
Schematic representation of inorganic solid electrolyte material classes.

Inorganic solid electrolytes (ISEs) are a class of ceramic materials that exhibit high ionic conductivity for lithium (Li), sodium (Na), or other alkali metal ions and can, therefore, provide a stable and efficient transport medium for ion flow between the anode and cathode in a battery [14]. While the use of ISEs is still relatively new and requires further research and development, it holds great potential to advance the field of energy storage and pave the way for safer, more efficient, and environmentally friendly batteries. Based on anion chemistry, ISEs are divided into three classes: oxide-based, sulfide-based, and halide-based [19]. Figure 2 illustrates further sub-divisions within these divisions, indicating additional material classes that will be discussed in this review. Each class of materials has unique advantages and limitations that make them suitable for different battery applications.

#### 2.1.1. Oxide-Based ISEs

Ceramic oxide SSEs are divided into three classes: garnet-type, perovskite-type, and NASICON-type, a sodium superionic conductor [14]. These materials typically display exceptional thermal stability, substantial bulk Li^+^ conductivity (ranging from 10^−3^ to 10^−5^ S cm^−1^ at 25 °C), and impressive Young’s moduli (>150 GPa) [14]. However, incorporating them into SSBs proves challenging due to their inherent mechanical rigidity. Furthermore, their notable bulk electronic conductivity (10^−8^ to 10^−7^ S cm^−1^) might inadvertently promote the formation of Li dendrites at the interface between Li and the solid electrolyte, as well as the growth and penetration of dendrites along grain boundaries [20].

Since 1981, when Weppner et al. discovered that Li_5_La_3_M_2_O_12_ (M = Ta or Nb) showcased an ionic conductivity of 10^−6^ S cm^−1^ at room temperature [21], there has been extensive exploration into garnet solid electrolytes. Some of the most explored materials of garnet-type SSEs are LATP and lithium lanthanum zirconate (LLZO).

LLZO is a ceramic material with a garnet crystal structure composed of Li, lanthanum (La), zirconium (Zr), and oxygen atoms. Its chemical formula is Li_6.4_La_3_Zr_1.4_Ta_0.6_O_12_. LLZO has a cubic garnet structure consisting of a network of ZrO_6_ octahedra and Li/La ions. The ZrO_6_ octahedra are arranged in a three-dimensional framework, with Li/La ions occupying interstitial sites between the octahedra [9]. Li^+^ can move through the interstitial sites to conduct current. LLZO is considered a promising SSE material due to several sophisticated inherent characteristics: its notable Li-ion conductivity reaching up to 10^−3^ S cm^−1^ at room temperature (RT) [22,23], comparably low electronic conductivity of approximately 10^−8^ S cm^−1^ (RT) [24], an expansive electrochemical stability window (>6 V vs. Li^+^/Li as observed in experiments) [25], as well as remarkable thermal and chemical stability in the presence of metallic Li. Due to these favorable attributes encompassing ionic conductivity, energy density, chemical stability, electrochemical stability, air stability, thermal stability, and safety, LLZO garnet-based electrolytes are widely regarded as one of the most promising and indispensable options [14,26]. However, LLZO suffers from manufacturing concerns, notably the cost of the required sintering techniques and the reproducibility of the LLZO microstructure during the sintering process [27].

LATP is a solid electrolyte material commonly used in SSBs due to its high ionic conductivity, chemical stability, and low reactivity with Li metal anodes [28]. LATP is a ceramic material with a garnet crystal structure composed of Li, aluminum (Al), titanium (Ti), phosphorus (P), and oxygen atoms. Its chemical formula is Li_1+x_Al_x_Ti_2-x_(PO_4_)_3_, where x is typically between 0.2 and 0.5. LATP has a complex crystal structure consisting of alternating layers of Li/Ti tetrahedra and Al/PO_4_ octahedra. The Li/Ti tetrahedra are connected by shared vertices to form a three-dimensional framework, while the Al/PO_4_ octahedra fill in the spaces between the tetrahedra [28]. This structure creates channels through which Li ions can move. LATP can be synthesized through a variety of methods, including solid-state reactions, sol-gel processes, and hydrothermal methods. In one common approach, lithium carbonate, aluminum oxide (Al_2_O_3_), titanium oxide (TiO_2_), and ammonium dihydrogen phosphate are mixed in stoichiometric proportions and heated at high temperatures (typically 900–1200 °C) in a reducing atmosphere to form the LATP ceramic [29]. Some advantages of LATP include a relatively high ionic conductivity, a wide electrochemical stability window, good mechanical stability, compatibility with Li metal anodes, low reactivity with cathode materials, and a wide temperature range. Additionally, LATP has a low tendency to form dendrites, which can improve the safety and cycle life of the battery. However, LATP is relatively expensive and has a lower ionic conductivity than LLZO [29,30].

While both materials have high ionic conductivity and good stability, there are some advantages of LLZO over LATP. LLZO is more chemically stable than LATP, particularly in the presence of moisture and air [26]. This stability can reduce the risk of degradation and improve the overall performance and reliability of the battery [9,10]. LLZO also offers lower reactivity with Li metal anodes compared to LATP, which can reduce the risk of dendrite formation [26]. Additionally, LLZO has higher mechanical strength than LATP, which can improve the durability and reliability of the battery. This mechanical strength is due to the crystal structure of LLZO, which is more robust than that of LATP. LLZO has better thermal stability than LATP, which means it can operate over a wider temperature range without degrading or breaking down. Finally, LLZO can be processed at a lower temperature than LATP, reducing the cost and complexity of manufacturing the electrolyte [9,10]. Despite these numerous advantages, LLZO is relatively expensive to produce and can have some processing difficulties due to its complex crystal structure [27].

The perovskite SSE commonly displays a chemical composition represented as ABO_3_, which falls within the cubic crystal system. Perovskite structures are typically characterized by notable Li^+^ conductivities ranging from 10^−3^ to 10^−4^ S cm^−1^ at RT [31,32], as well as a diminished electronic conductivity (~10^−8^–10^−9^ S cm^−1^) [32,33]. Lithium lanthanum titanium oxide (LLTO, La_2/3−x_Li_3x_TiO_3_) is an oxide-based ISE of perovskite-type and is the fastest Li^+^ conducting electrolyte of this type. It comprises Li, La with La-rich and La-poor domains, and vacancies within A sites, as well as Ti ions occupying B sites arranged in octahedral coordination with oxygen. LLTO can be synthesized through various methods, with solid-state reactions and sol-gel processes being the most common approaches. The specific synthesis method chosen can influence the properties of the resulting LLTO material [15]. LLTO boasts numerous advantages, including its substantial ionic transference numbers ranging from 0.5 to 0.9, remarkable chemical and thermal stability even in ambient air conditions, and its eco-friendliness due to the absence of toxic gas emissions during decomposition reactions [34]. Moreover, LLTO ISEs exhibit a broad electrochemical window spanning 8 V vs. Li/Li^+^ [31], enhancing their compatibility with high-voltage cathode materials and Li metal anodes [14]. Furthermore, LLTO showcases exceptional thermal stability across a wide temperature range (4–1600 K), expanding its potential applications even under extreme operational conditions [35]. However, several challenges hinder the seamless integration of LLTO ISEs into batteries. The presence of significant grain boundary resistance results in a total ionic conductivity that falls below 10^−5^ S cm^−1^ at room temperature [31,36]. Additionally, direct contact between LLTO and Li metal triggers chemical instability, as Li^+^ intercalation into LLTO at voltages under approximately 1.8 V causes a reduction of Ti^4+^ ions and escalated electronic conductivity [37]. The inherent brittleness of LLTO poses difficulties during the fabrication and assembly of battery components. Furthermore, the dynamic volume changes within batteries during operation can lead to delamination between ceramic oxide electrode and electrolyte layers, thereby curtailing the overall battery lifespan [31].

In 1976, through the high-temperature solid-state reaction approach, Goodenough et al. [38] created the solid electrolyte Na_3_Zr_2_PSi_2_O_12_ of the NASICON-type. Subsequently, the NASICON framework saw the derivation of a Li-ion SSE, LiA_2_(BO_4_)_3_, where the original Na^+^ ions were substituted with Li^+^ ions, with possible elements including A = Ti, Zr, Ge, or V, and B = P, Si, or Mo. Its Li counterpart, LiZr_2−x_Ti_x_(PO_4_)_3_, was synthesized by Subramanian in 1986 [38]. LISICON (lithium superionic conductor) represents a group of SSE materials crucial for advanced energy storage systems. LISICON materials comprise elements like Li, oxygen, and often silicon (Si), sulfur (S), or P, and offer remarkable Li^+^ conductivity, vital for efficient ion movement within batteries. LISICON is a Li^+^ conductor with the general formula Li_2+2x_Zn_1−x_GeO_4_ [39]. The synthesis of LISICON involves solid-state reactions at elevated temperatures, yielding a crystalline structure that delivers numerous benefits. LISICON’s advantages encompass high Li-ion conductivity even at room and elevated temperatures, ensuring efficient battery function. Moreover, these SSEs enhance battery safety by mitigating leakage, thermal runaway, and dendrite formation that can lead to short circuits. By enabling higher energy density, LISICON paves the way for batteries with extended charge-holding capacities. Its chemical and electrochemical stability contributes to battery longevity, and its adaptability to diverse temperature ranges makes it versatile for various applications.

However, LISICON does pose challenges. While oxide-derived LISICON materials exhibit notable Li^+^ conductivity, particularly under elevated temperatures, their conductivity values at RT are notably lower compared to sulfide-based LISICONs, as well as other oxide systems characterized by structures like NASICON, perovskite, and garnet [40]. Its intricate synthesis demands precise control of reaction parameters, possibly limiting large-scale production. Achieving the optimal balance of chemical composition and crystal structure for desired properties presents material-related difficulties. Additionally, the cost of manufacturing LISICON materials can be a concern, potentially influencing overall battery costs. Ensuring compatibility between LISICON electrolytes and electrode materials is crucial for ideal battery performance and longevity. While LISICON materials hold immense potential, their commercial use remains relatively limited due to ongoing research and development efforts and the need to address these challenges [40].

#### 2.1.2. Sulfide-Based ISEs

Sulfide solid electrolytes are a subclass of ISEs obtained by substituting oxygen atoms with S atoms in inorganic oxide solid electrolytes [41]. Sulfide electrolytes have garnered significant interest due to their exceptional Li^+^ conductivities exceeding 10^−4^ S cm^−1^, which can exceed that of organic liquid electrolytes [42,43,44,45]. Their favorable mechanical softness also facilitates excellent interactions with electrode materials [46]. Sulfide solid electrolytes can be divided into glass sulfide, glass–ceramic sulfide, and crystalline sulfide [14].

Lithium thiophosphate (LPS), with the chemical formula Li_2_S-P_2_S_5,_ is a notable glass-sulfide-based ISE of the argyrodite structure with properties that make it a promising candidate for SSB applications. Synthesized typically through solid-state reactions, LPS is produced by carefully mixing lithium sulfide (Li_2_S) and phosphorus pentasulfide (P_2_S_5_) in specific ratios, followed by heating to promote chemical reactions and crystal growth [47].

LPS offers several distinct advantages as a solid electrolyte material. Its high ionic conductivity (up to 10^−2^ S cm^−1^ at RT), particularly for Li^+^, allows for efficient ion transport within the solid electrolyte, enabling rapid charging and discharging in batteries [47]. Additionally, LPS is stable against Li metal, which is essential for mitigating safety risks associated with dendrite formation. Its compatibility with various cathode and anode materials enhances its versatility in accommodating different battery chemistries [48].

However, challenges also exist. The synthesis process requires careful control to achieve desired material properties, and the associated costs can impact the overall economics of the battery. Ensuring a stable interface between LPS and electrode materials remains a concern for long-term battery performance [49]. Mechanical properties, including brittleness, can pose manufacturing and operational challenges. Of utmost significance is that LPS is sensitive to both moisture and oxygen, introducing complexity to its processing when conducted under typical ambient air conditions [47].

One frequently employed glass-ceramic electrolyte is the (100 − x)Li_2_S-xP_2_S_5_ system, known for its superior ionic conductivity when contrasted with glass sulfide counterparts. In this system, the composition can vary by adjusting the value of x. It allows for a range of compositions where the ratio of Li_2_S to P_2_S_5_ can be controlled to fine-tune the properties of the glass-ceramic electrolyte.

Within the realm of crystalline-sulfide ISEs, two classes of materials are defined based on their structure: thio-LISICON (Li_10_MP_2_S_12_, M = Ge, Si, Sn) [50] and the argyrodite [14]. Within the thio-LISICON structure, two typical materials are often studied: LGPS and Li_9.54_Si_1.74_P_1.44_S_11.7_Cl_0.3_ [51].

LGPS is a highly promising solid electrolyte material renowned for its exceptional ionic conductivity, particularly at RT. LGPS reaches 1.2 × 10^−2^ S cm^−1^, higher than most organic liquid electrolytes [52]. Synthesized through solid-state reactions, LGPS is produced by meticulously blending precursor materials such as Li_2_S, germanium sulfide (GeS_2_), and P_2_S_5_ in precise proportions, followed by elevated-temperature heating to encourage chemical reactions and crystal growth [53,54]. The resultant LGPS material is ground into a powder for further processing [14].

The advantages of LGPS are manifold. Its high ionic conductivity facilitates rapid ion transport within the solid electrolyte, enabling efficient battery charging and discharging. Importantly, LGPS remains stable in the presence of Li metal, establishing a reliable interface and reducing the formation of potentially hazardous dendrites, thereby enhancing overall battery safety [55]. Its broad electrochemical stability window allows operation at higher voltage ranges, contributing to higher energy density batteries. LGPS’s compatibility with various cathode and anode materials adds to its versatility, catering to diverse battery chemistries. Moreover, SSBs that incorporate LGPS as the electrolyte exhibit enhanced safety by eliminating flammable liquid electrolytes, mitigating the risks of fire and explosion under extreme conditions.

However, LGPS does present certain challenges. Its synthesis entails energy-intensive high-temperature processes, necessitating careful control to achieve desired material properties. The associated costs, both in terms of synthesis and materials, could impact the overall affordability of batteries. While LGPS itself is stable against Li metal, achieving a consistently stable interface between the solid electrolyte and electrode materials remains a hurdle [56,57,58]. Interface reactions could influence long-term battery performance and cycling stability [59]. Moreover, LGPS and similar solid electrolyte materials can be brittle with inferior mechanical properties, potentially resulting in issues like cracking or delamination during battery fabrication and operation [60]. Additionally, the hygroscopic nature of LGPS requires meticulous handling and storage practices to prevent moisture absorption, which could compromise its performance.

Li_9.54_Si_1.74_P_1.44_S_11.7_Cl_0.3,_ a solid electrolyte material, offers promising advantages and faces notable disadvantages in the context of LIBs. On the positive side, it exhibits high Li^+^ conductivity (2.5 × 10^−2^ S cm^−1^), enabling faster charging and discharging, potentially reducing charging times [61,62]. Solid electrolytes like this one are known for their enhanced safety compared to liquid counterparts, as they are less prone to leakage and thermal runaway. They also operate effectively across a wide temperature range and can be compatible with high-capacity anode materials, which may lead to batteries with higher energy density and longevity, while reducing the formation of Li dendrites [63].

However, the commercial availability of Li_9.54_Si_1.74_P_1.44_S_11.7_Cl_0.3_ and similar solid electrolyte materials remains limited, hindering immediate widespread adoption. Manufacturing challenges, including complexity and cost, must be addressed to scale up production for broader use [64]. Some solid electrolytes may also exhibit mechanical stability issues and interface compatibility concerns with other battery components [50,65]. Finally, the cost of production for these materials may be higher than traditional liquid electrolytes, impacting the overall cost of LIBs. Despite these challenges, ongoing research and development efforts aim to maximize the advantages while mitigating the disadvantages, potentially revolutionizing the future of battery technology.

#### 2.1.3. Halide-Based ISEs

While oxide and sulfide-based electrolytes are typically the most studied, there is still investigation into halide-based ISEs. The latter are a subset of solid electrolyte materials that incorporate halide ions (e.g., fluoride, chloride, bromide, and iodide) as part of their composition. Compared to oxide and sulfide-based ISEs, halide ISEs exhibit a more well-rounded set of properties across different factors, encompassing ionic conductivity, electrochemical stability window, and moisture resistance. These materials have gained attention for their potential use in SSBs due to their high ionic conductivity and compatibility with various battery chemistries [13]. However, halide-based solid electrolytes can also present challenges related to stability and materials processing, specifically due to their sensitivity to moisture [66].

Halide-based SSEs offer a diverse landscape, classifiable into three distinct categories, each exhibiting unique characteristics and potential applications. The first class encompasses Li_3_MX_6_ halide electrolytes, where M represents group 3 elements such as scandium (Sc), yttrium (Y), and various lanthanides. The second class involves Li_3_MX_6_ halide electrolytes incorporating group 13 elements such as Al, gallium (Ga), and indium (In). Finally, the third class consists of Li_2_MX_4_ or Li_6_MX_8_ halide electrolytes that involve divalent metal elements, including Ti, zirconium (Zr), hafnium (Hf), vanadium (V), chromium (Cr), manganese (Mn), iron (Fe), zinc (Zn), and magnesium (Mg) [67]. Some of the most extensively studied halide electrolytes are Li_3_YCl_6_, Li_3_ScCl_6_, and Li_3_YBr_6_ [68]. While halide SSEs continue to be explored, oxide and sulfide materials currently dominate the field of SSE research.

### 2.2. Organic Solid Polymer Electrolytes (OSPEs)

Polymer electrolytes have emerged as a promising alternative to traditional ISEs in SSBs due to their unique properties and potential advantages. Unlike inorganic electrolytes, polymer electrolytes are made of organic polymers that can be designed to have high ionic conductivity, good thermal stability, and mechanical flexibility. Additionally, polymer electrolytes can reduce the interface resistance between the electrodes and the electrolyte, improving battery performance. Furthermore, polymer electrolytes can be processed using cost-effective and scalable methods, making them attractive for large-scale manufacturing. Despite these promising characteristics, polymer electrolytes face challenges related to low ionic conductivity, chemical stability, and mechanical strength [69,70]. Therefore, ongoing research is focused on developing new polymer materials and optimizing the properties of existing ones to overcome these limitations and unlock the full potential of polymer electrolytes in SSBs.

PVDF is a type of polymer material that is sometimes used as an electrolyte in SSBs, particularly in combination with Li salts such as lithium bis(trifluoromethanesulfonyl)imide (LiTFSI) [69,71]. PVDF is a polymer composed of carbon, hydrogen, fluorine, and sometimes other elements such as oxygen or chlorine. Its repeating unit is CH_2_CF_2_, and the polymer chain can be linear or branched depending on the specific polymerization process used. PVDF can be synthesized through a process known as polymerization, in which monomers such as vinylidene fluoride are reacted in the presence of a catalyst and/or initiator to form a polymer chain [72]. The resulting PVDF polymer can be further processed into various forms, such as films, fibers, or powders.

To use PVDF as an electrolyte in SSBs, the polymer is typically combined with a Li salt such as LiTFSI. The PVDF/LiTFSI mixture can be dissolved in a solvent such as acetonitrile or propylene carbonate to form a gel or polymer electrolyte. The resulting electrolyte can be cast into films or other shapes and incorporated into the battery design [72]. PVDF-based electrolytes have some advantages over other types of solid electrolytes. They can have relatively high ionic conductivity and good mechanical properties, which can improve the overall performance and stability of the battery [8]. However, PVDF-based electrolytes can also have drawbacks, such as limited electrochemical stability and potential reactivity with Li metal anodes [70]. As a result, PVDF-based electrolytes may be more suitable for specific battery designs or applications rather than being a universal solution.

PEO is a polymer material commonly used as an electrolyte in SSBs, particularly in combination with Li salts such as LiTFSI. PEO is a polymer composed of carbon, hydrogen, and oxygen. Its repeating unit is CH_2_CH_2_O, and the polymer chain can be linear or branched depending on the specific polymerization process used [70]. PEO-based electrolytes have some advantages over other types of solid electrolytes. They can have relatively high ionic conductivity and good mechanical properties, which can enhance the overall efficiency and durability of the battery. PEO-based electrolytes also have good compatibility with Li metal anodes, which can reduce the risk of dendrite formation and improve the overall safety of the battery [71]. In addition, PEO-based electrolytes can be relatively low-cost and easy to manufacture compared to other solid electrolytes [73]. However, PEO-based electrolytes can also have some drawbacks. They can have limited electrochemical stability and be prone to degradation over time, particularly in the presence of moisture or other contaminants. In addition, PEO-based electrolytes can be relatively sensitive to temperature and may require careful control of the operating conditions to maintain their performance [71,73].

Poly(acrylonitrile) (PAN) is a polymer that has been investigated as a potential electrolyte material for SSBs. PAN-based polymer electrolytes have been shown to have high ionic conductivity and good mechanical properties, which make them attractive for use in SSBs [73]. The composition of PAN-based polymer electrolytes typically involves mixing PAN with a Li salt and a plasticizer, which helps improve the polymer electrolyte’s ionic conductivity. The Li salt dissociates in the polymer matrix to form free Li^+^, which is responsible for the charge transport within the electrolyte.

PAN-based polymer electrolytes can be manufactured using cost-effective and scalable methods, such as solution casting or electrospinning. Solution casting involves dissolving PAN, the Li salt, and the plasticizer in a solvent and casting the resulting solution into a thin film. Electrospinning consists of using an electric field to spin a polymer solution into nanofibers, which can be used to form a three-dimensional network that enhances the mechanical strength and ionic conductivity of the electrolyte [74]. One advantage of PAN-based polymer electrolytes is their high ionic conductivity, which can be attributed to the dissociation of the Li salt and the plasticizer’s ability to increase the mobility of the Li^+^. PAN-based polymer electrolytes also have good mechanical properties, such as high elasticity and tensile strength, which make them resistant to deformation and cracking during battery operation.

Overall, PAN-based polymer electrolytes show promise as a potential electrolyte material for SSBs. Ongoing research is focused on optimizing the composition and processing of PAN-based polymer electrolytes to improve their ionic conductivity, mechanical properties, and stability over the battery’s lifetime. The ionic conductivities of some notable ISEs and OSPEs are shown in Table 1.

### 2.3. Composite Solid Electrolytes (CSEs)

**Figure 3:**
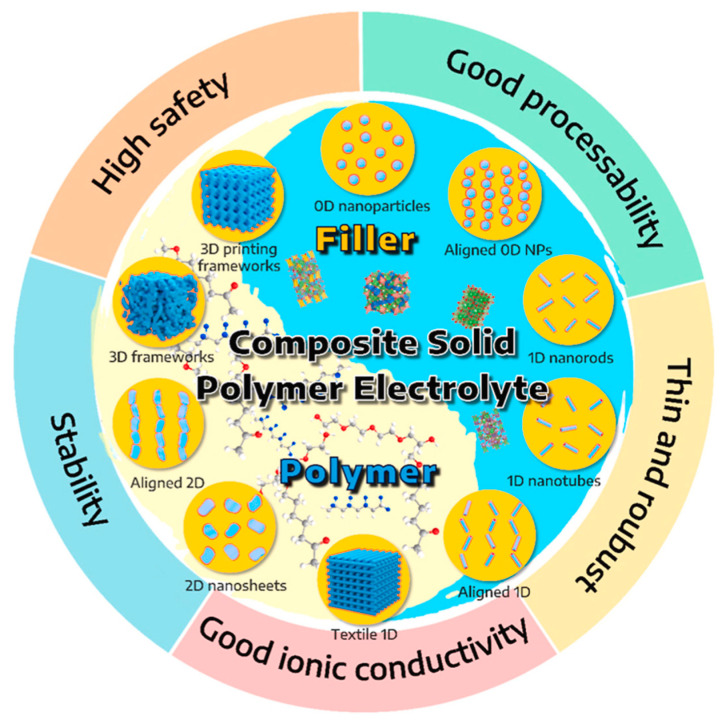
Illustration depicting the structure of fillers and template polymers used in developing CSEs. The graphic also highlights the overarching benefits of using CSEs [15]. Reprinted with permission from Elsevier.

While some studies have concentrated on either inorganic solid ceramic electrolytes or organic solid polymer electrolytes, there is a rising trend of research attention towards CSEs. These electrolytes merge the strengths of both inorganic and organic solid electrolytes while eliminating their drawbacks. In CSEs, inorganic ceramic electrolytes function primarily as fillers to improve mechanical strength and ionic conductivity [74]. Some examples of these fillers and the overall advantages of CSEs are shown in Figure 3.

The purpose of adding inorganic fillers into the polymer matrix is to improve mechanical strength, increase ionic conductivity, and improve stability. Recent research includes the investigation of various morphologies such as 0D nanoparticles, 1D nanowires, 2D nanosheets, and 3D frameworks. Depending on the Li ion conductivity, the inorganic fillers can be classified into two categories: passive and active.

Passive inorganic fillers are commonly used in polymer composite SSEs to improve their mechanical and thermal properties. These fillers do not participate in the ionic conduction process but rather act as a supporting material to enhance the overall performance of the composite electrolyte. Inert fillers are mainly oxide ceramics with a spherical particle shape, like Al_2_O_3_, silica (SiO_2_), and TiO_2_ [74,76].

One common passive filler used in polymer composite SSEs is SiO_2_, known for its excellent mechanical properties and thermal stability. Adding SiO_2_ nanoparticles to polymer electrolytes has been shown to improve their mechanical strength, modulus, and thermal stability while maintaining their high ionic conductivity. In terms of electrochemical properties, studies have shown that adding SiO_2_ nanoparticles can improve the ionic conductivity of polymer electrolytes. This is thought to be due to the increase in the number of ion-conducting pathways within the composite electrolyte, as well as the improved interfacial contact between the polymer matrix and the SiO_2_ nanoparticles.

A recent study found that by adding SiO_2_ nanotubes to PEO/LiTFSI, the ionic conductivity increased from 6.13 × 10^−8^ S cm^−1^ to 4.35 × 10^−4^ S cm^−1^ at 30 °C [77]. They proposed that the interaction between the SiO_2_ nanotubes and the composite contributed to the efficient transport of Li^+^. Additionally, the assembled cell showed good cycle life. Other passive fillers that have been investigated include Al_2_O_3_, magnesium oxide (MgO), and titanium dioxide (TiO_2_). These fillers have been shown to improve the mechanical and thermal properties of the polymer electrolytes while maintaining their high ionic conductivity [78].

Incorporating passive inorganic fillers into polymer composite SSEs can provide several benefits, such as improved mechanical strength, better thermal stability, and increased resistance to deformation and cracking. These benefits can make the composite electrolytes more durable and longer lasting in demanding applications, such as high-performance LIBs or supercapacitors.

Active fillers contain Li ions in their composition and are used in polymer composite SSEs to enhance their ionic conductivity by providing a continuous pathway for ion transport. These fillers are typically ceramic materials with high ionic conductivity and can act as an active component in the composite electrolyte.

One of the most common active inorganic fillers used in polymer composite SSEs is Li-ion conducting ceramics, such as LLZO, LATP, and lithium phosphorus oxynitride (LiPON). These materials have high ionic conductivity and can provide a continuous pathway for ion transport in the composite electrolyte, resulting in higher overall ionic conductivity. Recent research has shown that incorporating LLZO into polymer electrolytes can significantly improve their ionic conductivity. For example, one study found that a composite electrolyte containing 30 wt.% LLZO achieved an ionic conductivity of 2.2 × 10^−4^ S cm^−1^ at room temperature, much higher than that of the pure polymer electrolyte. Researchers have also investigated the effect of different types of LLZO particles on the performance of composite electrolytes and found that smaller LLZO particles with a higher surface area led to higher ionic conductivity [79]. Recent studies have found that LATP can improve the ionic conductivity of polymer electrolytes, as well as their thermal stability and mechanical strength. For example, one study found that a composite electrolyte containing a porous LATP framework was able to serve as a physical barrier to suppress the growth of Li dendrites and showed an ionic conductivity of 7.47 × 10^−4^ S cm^−1^ at 60 °C, which is higher than that of PEO (1.0 × 10^−4^ S cm^−1^) at RT [80].

NASICON is an active inorganic filler material with great potential for improving the ionic conductivity of polymer composite SSEs. Recent research has focused on optimizing the use of NASICON as an active filler material. One study found that incorporating NASICON into a polymer electrolyte significantly improved its ionic conductivity and mechanical properties. The researchers found that the optimal NASICON content was 20 wt.%, which resulted in a polymer electrolyte with a high ionic conductivity of 1.44 × 10^−3^ S cm^−1^ and good mechanical strength. Another study investigated the effect of sodium doping on the ionic conductivity of NASICON-based SSEs. The researchers found that increasing the amount of sodium doping led to an increase in the ionic conductivity of the material. They also found that the addition of NASICON improved the thermal stability of the polymer electrolyte, making it more suitable for high-temperature applications [81].

Other active fillers that have been investigated include sulfides, oxides, and nitrides, such as Li_2_S, lithium nitride (Li_3_N), and lithium magnesium oxide (LiMg_0.05_O). These materials have also been shown to improve the ionic conductivity of the polymer electrolyte and enhance its overall performance. Compared with passive fillers, active fillers have a stronger enhancement effect on the ionic conductivity of SPEs. This is mainly due to the intrinsic high bulk ionic conductivity of active ceramics [76]. Examples of some CSEs, along with their ionic conductivity, are shown in Table 2.

## 3. Electrode Materials for SSBs

### 3.1. Anode

The anode is the battery’s negative electrode and is responsible for releasing electrons during the discharge process. In SSBs, the anode is typically made of a Li-containing material, such as Li metal, lithium titanium oxide (Li_4_Ti_5_O_12_), or lithium silicon (LiSi). These materials are chosen because they have high energy densities and good stability but can also be prone to degradation over time [88]. Substituting the traditional graphite anode with Li metal presents a promising avenue. Indeed, according to Jie Xiao [89], this could amplify the energy density of batteries by a factor of approximately 1.5. This transition, however, sets in motion a series of chemical alterations within the liquid electrolyte upon encountering Li metal and is, therefore, only possible with a solid electrolyte. Indeed, these transformations give rise to the creation of hazardous organic salts, which subsequently precipitate and evolve into the infamous structures known as Li dendrites. These dendritic formations can grow and extend, ultimately piercing through the separator that keeps the battery’s components isolated. This breach in the separator leads to short circuits within the battery, thereby introducing a significant safety concern [89].

Nonetheless, the utilization of solid electrolytes brings forth a set of distinctive advantages. These SSEs possess characteristics that render them non-volatile and non-flammable, making the battery safer. Moreover, they exhibit an extended range of electrochemical stability, allowing for more efficient battery operation. Critically, they offer the capability to effectively inhibit the progress of Li dendrites. Indeed, Pilgun et al. [90] showed that by preventing these dendrites from penetrating the separator, the solid electrolytes play a pivotal role in enhancing the overall safety of the battery.

Thus, SSEs allow the employment of metallic Li as a negative electrode. Currently, most SSBs opt for a Li metal anode due to its promising theoretical capacity, lightweight nature, and low electrochemical potential. However, Li has drawbacks like high reactivity, susceptibility to oxidation, and limited availability compared to potential substitutes. Moreover, there have been instances of Li dendrite formation, even when using certain SSEs, which poses safety concerns for the battery. Consequently, researchers are actively exploring alternative anode materials such as Si, S, metallic alloys, tin (Sn), Ti, and carbon-based substances [91]. This subsection will delve into these diverse options, but first, attention is given to lithium-based anodes.

Li metal is commonly used as an anode in SSBs due to its high theoretical capacity, lightweight nature, and low electrode potential. However, it also has drawbacks related to its high reactivity, susceptibility to oxidation, and dendrite formation, posing safety concerns. Researchers have improved the electrochemical performance of these systems by using graphite coatings, facilitating three-dimensional Li-ion transport on the graphite’s surface and enhancing mechanical properties [92].

Another option is lithium titanate (LTO, Li_4_Ti_5_O_12_) anodes. They are known for their safety and cycle life and, although not offering the high energy density of Li metal, they are stable and dependable. Garnet-structured SSEs are gaining attention, but their lithiophobic nature creates interface resistance. A composite anode material comprising Li metal and LTO has been proposed to address this challenge [93].

Silicon stands out as a highly promising anode material for batteries due to its exceptional theoretical capacity, cost-effectiveness, and stability in air. It has garnered significant attention for use in EVs and SSBs, addressing concerns related to energy storage and driving range limitations in the EV industry [94]. However, Si faces inherent challenges, notably substantial volume expansion and pulverization during charge and discharge cycles. Researchers are actively exploring innovative solutions to mitigate these issues, aiming to enhance the performance and longevity of Si-based battery systems [94]. In the context of SSBs, integrating Si-based negative electrodes is a key focus to align with advanced electrolyte technologies and establish stable battery operation [94]. Nevertheless, the relatively low ionic conductivity of solid electrolytes and the substantial resistance encountered at the electrode-electrolyte interface present challenges that affect overall battery performance. The appeal of Si as an anode material in ASSBs stems from its ready availability, non-toxic nature, and remarkable theoretical capacity, making it a competitive candidate in the pursuit of next-generation battery technology [95]. However, it is essential to acknowledge certain limitations associated with Si-based anodes, including concerns related to mechanical integrity, limited electrical conductivity, and cycling lifespan [96,97]. These challenges underscore the ongoing efforts in material modification and engineering to harness Si’s full potential while addressing its drawbacks in advanced battery systems.

Lithium silicides (Li-Si alloys) and sulfur are other potential anode materials. They offer higher energy density but face challenges such as low electrical conductivity. To enhance the energy storage capability, researchers have explored heteroatom doping, including S, P, nitrogen, oxygen, and boron doping. Among these, S doping has garnered significant attention. S atoms have a larger covalent radius, which expands the interlayer spacing of carbon materials, thereby increasing the number of active sites available for sodium storage [98].

Certain SSBs are exploring metallic alloys like lithium-aluminum (Li-Al) and lithium-tin (Li-Sn), striking a balance between capacity and stability [99]. In a study by Zhang et al., a polymer electrolyte reinforced with polyacrylonitrile (PAN) fibers and a protective Li-Sn alloy layer on the Li anode has significantly extended the cycle lifespan of room-temperature SSBs. The Li-Sn alloy layer acted as a passivation layer, preventing unwanted reactions between metallic Li and the solid polymer electrolyte (SPE), and enhancing compatibility and stability [100]. Alloy anodes in SSBs offer mechanical advantages over other materials, avoiding issues like short-circuiting and stabilizing the solid-electrolyte interphase, thus advancing the development of efficient and reliable SSBs [101].

Finally, silver-carbon composite interlayers have shown potential for enabling Li-free cycling in SSBs. During battery charge, Li intercalates into graphite and reacts with Ag to form Li-Ag alloys. Discharge proceeds through Li-deficient Li-Ag phases. Higher charging rates delay Li-Ag phase formation, resulting in more Li metal deposition [102].

### 3.2. Cathode

The cathode is the positive electrode of the battery, responsible for accepting electrons during the discharge process. In SSBs, the cathode is typically made of a Li-containing material, such as lithium cobalt oxide (LiCoO_2_), lithium iron phosphate (LiFePO_4_), or lithium nickel manganese cobalt oxide (LiNiMnCoO_2_). These materials are chosen because they have high energy densities, good stability, and relatively low cost [88], but can only be properly used in non-reactive electrolytes. Indeed, some high-energy cathode materials, such as those based on nickel or manganese, can be sensitive to the electrolyte’s chemistry. SSEs can provide a more stable interface with these cathode materials, reducing unwanted side reactions and enhancing the overall efficiency and cycle life of the battery [103]. Moreover, cathodes that interact directly with air or oxygen, such as lithium-air (Li-O_2_) or sodium-oxygen (Na-O_2_) batteries, can benefit from SSEs that prevent the infiltration of moisture and contaminants from the air [104,105]. It extends the lifespan of the cathode by inhibiting dendrite growth and unwanted reactions. The wider electrochemical stability window allows higher voltage operation, potentially boosting energy density. Additionally, the SSE prevents cathode-electrolyte reactions, preserving cathode capacity. Its stability enhances safety for thermally sensitive cathodes, and its interface with the cathode improves overall electrochemical performance. Overall, SSEs provide flexibility in cathode selection, minimize self-discharge, and positively impact the cathode’s stability and performance over time [14].

However, the primary determinant of battery energy density remains the selection of cathode materials. This choice significantly impacts the potential energy storage within the battery. To achieve heightened energy density, it becomes crucial to diminish the resistance at the interface connecting the electrolyte and the electrode [106].

Unlike traditional LIBs, SSBs function in a distinct manner where the movement of Li^+^ occurs through the solid portions, encompassing both bulk and solid-to-solid interfaces. Consequently, optimizing the connections between SSEs and cathode materials becomes essential to establish efficient pathways for Li^+^ transport within the electrode [107]. The decision regarding the cathode material is thus heavily influenced by the chosen electrolyte. Their compositions must often be closely aligned to prevent additional complications at the interface.

For ISEs, cathode materials rich in nickel (Ni) have recently been used in LIBs due to their ability to hold high reversible capacities exceeding 200 mA g^−1^. Unfortunately, the applicability of these cathode materials in SSBs is limited due to their relatively low density, which can lead to particle cracking and loss of Li-ion transport pathways. This, in turn, results in notable performance degradation during the cycling of SSBs [108]. To address this issue, researchers have explored various structural adjustments to enhance the mechanical strength and density of cathode materials, aiming to make them compatible with SSBs. In this context, cathode materials with a single-crystalline structure show promise due to their cohesive form, absence of defects in their microstructure, and excellent particle hardness [106].

Research findings indicate that the interfacial resistance at the solid-solid junctions between the lithium phosphorus sulfur chloride (LPSCl) solid electrolyte and cathode materials can be effectively lowered by modifying the structure of Ni-rich cathode materials to adopt a single-crystalline form [106]. An effective technique to mitigate interfacial impedance involves applying a coating of Li ionic conductors onto oxide cathodes. This method has shown improvements in initial charge-discharge capacity and rate performance. For instance, LiNbO_3_-coated Ni-rich LiNi_0.8_Co_0.1_Mn_0.1_O_2_ (NCM811) cathode has exhibited noteworthy electrochemical enhancements in SSBs operating at 35 °C and 60 °C [109]. However, challenges remain regarding cycle performance enhancement for oxide cathodes, primarily due to issues like incomplete coating with thin inactive buffer layers or decreased electronic conductivity and specific capacity with thicker inactive buffer layers. To address this, constructing a core-shell architecture for Ni-rich oxide cathode materials proves effective, where the Ni-rich core contributes to high capacity, and the Ni-low shell ensures stable interactions with sulfide electrolytes, allowing for complete coating [108,109].

Zhang et al. [110] demonstrated that a solid-state lithium metal battery using a ceramic-based composite solid electrolyte and a LiNi_0.5_Co_0.2_Mn_0.3_O_2_ (NCM523)-based composite cathode yielded superior performance. Yu et al. [111] proposed hexaazatriphenylene (HATN)-based organic materials as suitable cathodes for quasi-solid-state lithium-organic batteries. Combining these organic cathodes with a gel polymer electrolyte modified with a succinonitrile plasticizer resulted in improved electrochemical performance.

Moreover, addressing resistance at the electrolyte-electrode interface is crucial to elevate energy density. Unlike conventional LIBs, SSBs function uniquely, necessitating optimized connections between solid electrolytes and cathodes for efficient Li ion transport. The choice of cathode material is intricately tied to the electrolyte, requiring compositional alignment to prevent complications. While ISEs have succeeded with nickel-rich cathodes in LIBs, their potential in SSBs is hindered by density-related challenges, prompting innovative solutions such as single-crystalline structures. Research shows that modifying cathode structures can reduce interfacial resistance, and strategies like Li ionic conductor coatings on oxide cathodes can significantly enhance performance. Organic-based cathodes, as exemplified by HATN, are especially promising when combined with advanced electrolyte modifications.

## 4. Additive Manufacturing of SSBs

SSBs are safer and better than LIBs and can be produced at a commercial scale by additive manufacturing, also known as 3-dimensional (3D) printing. Researchers and engineers have been exploring the integration of 3D printing techniques to fabricate SSBs, aiming to enhance their efficiency, energy density, and overall performance. The use of 3D printing in the fabrication of SSBs offers advantages such as intricate design possibilities, improved manufacturing precision, and the ability to create complex internal structures that enhance battery performance [112]. Researchers have investigated various 3D printing technologies, including selective laser sintering, stereolithography (SLA), and roll-to-roll printing, to create intricate solid electrolyte structures and optimize the overall battery architecture [113].

For electrode materials, conventional thin film fabrication methods, including sol-gel techniques, electron beam evaporation, and chemical vapor deposition, are complex and expensive, often leading to undesired side reactions that reduce the efficiency of LIBs. Inkjet printing, as reported by Zhao et al., offers a simpler approach, demonstrating improved electrochemical efficiency for SnO_2_ and LiCoO_2_ thin film electrodes [114]. Substituting conventional carbon black with surface-modified carbon in the ink further enhances electrochemical properties. Utilizing the direct ink writing (DIW) technique, planners and a 3D-patterned LiMn_2_O_4_ cathode were constructed, incorporating carbon black, PVDF, and N-methyl-2-pyrrolidone [115]. The resultant cell exhibited superior specific capacity and rate capability when compared to conventional flat electrodes.

Printing techniques are also being employed for the fabrication of SSEs. Delannoy et al. investigated LIB construction incorporating porous electrodes, where the SSE was fabricated using inkjet printing with a silica-based chemical solution as ink. The inkjet-printed electrolyte exhibited comparable electrochemical performance to the physically vapor-deposited counterpart [116]. In parallel, diverse 3D-printable LATP-based inks were developed through the DIW technique, enabling the creation of arbitrary shapes (L, T, and +) and achieving higher conductivities (up to 4.24 × 10^−4^ S cm^−1^). These inks were employed in the construction of ceramic SSEs. LATP-based electrolytes were directly printed on LiFePO_4_ cathodes, demonstrating a high discharge capacity of 150 mA h g^−1^ at 0.5 °C [117]. In a noteworthy contribution to overcoming the obstacle of poor interfacial contact within SSBs, a recent study reports the use of SPEs fabricated through SLA 3D printing for SSBs. The SLA-printed OSPE demonstrates an impressive ionic conductivity of 3.7 × 10^−4^ S cm^−1^ at 25 °C, as well as reduced interfacial impedance, outperforming structures utilizing conventional structure-free OSPE [118].

The integration of 3D printing can potentially streamline the manufacturing process of SSBs, reducing production costs and making these advanced energy storage devices more commercially viable [112]. As research and development in this field continue, it is anticipated that further breakthroughs will be made, pushing the boundaries of both 3D printing and SSB technology.

## 5. Solid-State Battery Market

### 5.1. Market Overview

**Figure 4:**
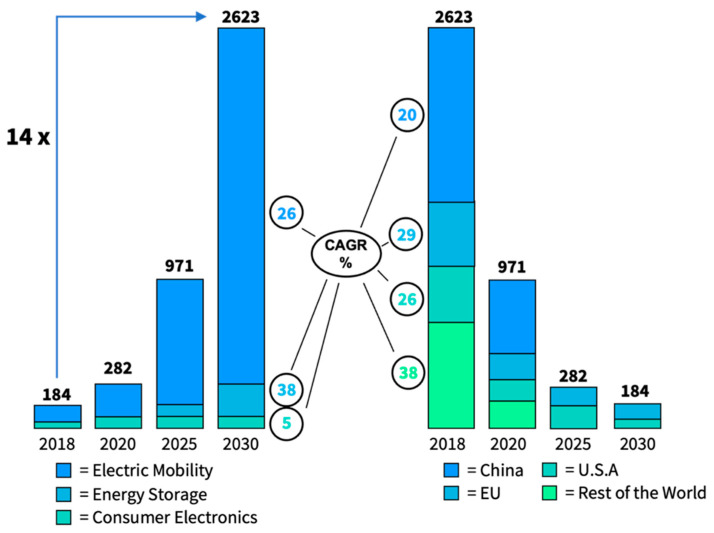
Global battery demand by application and region (in GWh) with respective compound annual growth rates (CAGRs, %) [121].

The World Economic Forum (WEF) forecasts the global battery market to grow from a capacity of ≈330 GWh in 2018 to ≈2.6 TWh in 2030, representing a 14-fold increase. Figure 4 shows that the bulk share of the demand originates from the electric vehicle (EV) mobility sector, with an expected compound annual growth rate (CAGR) of 26% [119]. Initial calculations for the year 2021 indicated a capacity of around 400 GWh. According to the Fraunhofer Institute ISI, the market has seen recent annual growth between 30% and over 40%, even faster than previously predicted by the WEF [120].

The global battery market is currently dominated by lead-acid (PbA) and LIBs. Overall, the global demand for LIBs was 250–280 GWh in 2020, with a market revenue of over 35 billion euros in cells sold, while current state-of-the-art cell costs are around 90 €/kWh [122]. Material costs typically make up the largest portion. Cathode costs, in particular, account for roughly half of these material costs. Costs associated with cell assembly are approximately at the 17 €/kWh benchmark. The main technology drivers include applications for EVs, portable consumer electronics, and future energy storage systems. Market drivers, such as regulations for emission control, the increasing renewable energy sources (RES) generation, and the necessary storage capacities, are also significant. Consequently, SSBs will directly compete with LIBs in the future. This market analysis will, therefore, systematically examine SSB market progress in relation to historical and future LIB technology advances. This is due to their technological proximity and the fact that many available data sources naturally highlight this relationship.

According to the Fraunhofer Institute ISI, global demand for LIBs could reach more than 3 TWh per year by 2030, with most reports and market forecasts predicting a global demand of 1–4 TWh for the year 2030 (maximum scenarios assume a demand of up to 6 TWh). This capacity would be equivalent to market revenues of 125–225 billion euros (assuming a price drop of 70–80%) [120]. Production line investments of 130 billion euros will be required globally until 2030. Next to this, new markets (individual passenger aviation and others) could reach a relevant market share by 2030, which will further increase demand. In the long term, beyond 2030, a global battery demand of more than 10 TWh per year is predicted [120].

Consumer concerns regarding EVs (short driving ranges and long charging times) stand out as the main market driver for battery improvement. This may be the reason why SSBs are on the roadmap of many battery producers and original equipment manufacturers (OEM), as the key performance indicators (KPI) of SSBs indicate great suitability for electric mobility [123]. EV-makers decide on mass commercialization of SSB and will be the decisive element for the organization of the supply chain, as they impose the requirements and make strategic decisions that affect the entire market [119].

### 5.2. SSB Market Size

The entire SSB market is generally an emerging type of market. The current utilization of SSB is limited to low-volume EVs and some smaller portable devices (sensors or medical applications). It almost exclusively consists of polymer-based SSBs, and in some cases, SSB microbatteries with oxide thin film electrolytes (e.g., solid batteries for medical devices manufactured for more than 15 years by companies like Ilika [124]). Apart from some exceptions, few of these SSB products have been on the market, such as an ultra-thin ceramic SSB from STMicroelectronics since 2014 [125]. The thin film battery technology employed for the small-scale applications differs significantly from the large-scale battery cells that are required for EV installations, both in terms of material and cell design, as well as manufacturing, so economic comparisons and conclusions from these cannot be applied to the general SSB future market.

As a mostly emerging technology, it must be emphasized in advance that SSB market size projections are subject to strong uncertainties given the small amount of freely available economic data. SSB’s global production capacity is estimated to be below 2 GWh, almost exclusively based on polymer SSBs [120]. This global capacity is equivalent to less than 0.5% of LIBs’ capacities. Based on the hypothesis that the main technological barriers will be solved, the prospected demand for SSB in EVs will rise from 200 MWh in 2022 to 2 GWh in 2025 [119], which is equivalent to a CAGR of 118%. It is important to approach this situation with caution when examining other sources, as most roadmaps or producers’ statements aim to start industrial-scale production of SSBs around 2025, as the earliest starting year of manufacturing [126].

**Figure 5:**
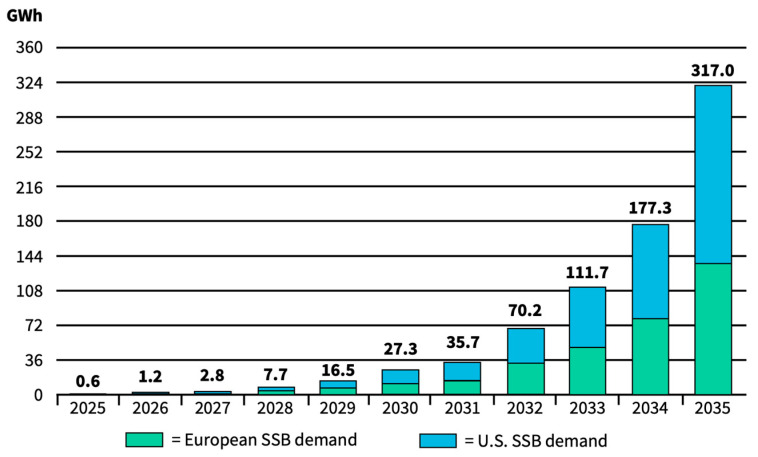
Forecasted SSB demand in Europe and the United States (in GWh) [127].

This capacity is anticipated to increase significantly, especially with the emergence of oxide and sulfide electrolyte-based SSBs in the market between 2025 and 2030. The total capacity of the SSB market is estimated to be 15–40 GWh in 2030 and 55–120 GWh in 2035, which is still relatively small compared to the total LIB market of 1–6 TWh around 2030 and 2–8 TWh by 2035 [120]. With SSB shares expected to rise from the current 0.5% to just above 1% by 2035, it must be emphasized that LIBs are set to dominate the global battery market for the foreseeable future. It is necessary to highlight the substantial deviations among different data sources. While the Fraunhofer Institute ISI puts forward a rather conservative growth expectation of SSBs in the range of 5–120 GWh by 2035, Bloomberg New Energy Finance (Bloomberg NEF) forecasts more than 300 GWh, while only taking the U.S. and Europe into account (Figure 5).

**Figure 6:**
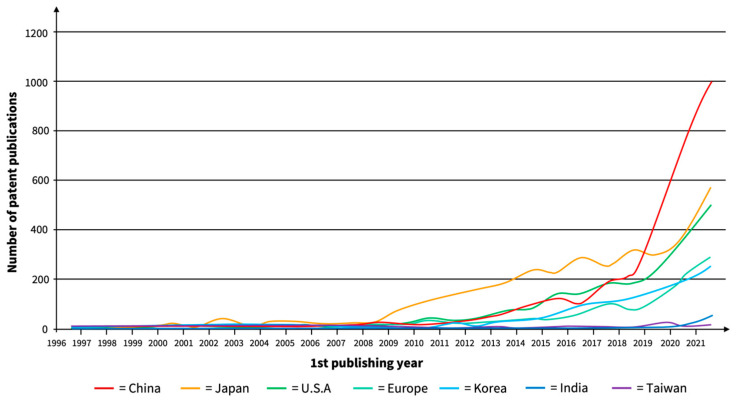
Evolution of SSB patent filings per country [130].

Regarding the revenue size projections of SSBs (as part of the global LIB market), it is still difficult to make projections for the future; however, they are significantly smaller than respective LIB sales. IdTechEx Research forecasts the SSE industry to reach a market size of over 25 billion euros by 2029 [128], and Lux Research expects the SSBs market to grow to 42 billion euros by 2035 [129]. While it is difficult to project the market valuation accurately, the growth forecasts and market dynamics can partly be validated by the intense patent activities relating to SSB research. In a highly dynamic and competitive environment, the patent landscape is still quantitatively dominated by Japanese companies from all over the value chain, with Japanese entities owning the great majority of enforceable Japanese patents [130]. Toyota, the world’s largest automotive manufacturer, holds over 1300 patents alone [131]. However, Chinese patent filing has accelerated explosively in recent years. The evolution of SSB patent filings is shown in Figure 6.

It can be assumed that the intense IP competition will lead to fast-tracked innovations and leapfrogging technological barriers. The close relationship between the capacity development forecasts and the number of patents filed reinforces the assumption that the market forecasts are realistic, with many R&D departments of leading battery and automotive manufacturers investing heavily in SSB research.

### 5.3. Economics of SSBs

Analogous to market predictions, expectations of the cost structure of SSBs also fluctuate. According to Allied Market Research, current SSB prices are estimated to range from 400 to 800 €/kWh by 2026 [132]. While most technologies are expected to enter markets at still high prices, exceptions exist. The Silicon Valley-based SSB start-up “Ampcera” announced a 75 €/kWh SSB in 2021, with energy densities higher than 450 kWh/kg [133]. Generally, optimistic price values must be taken with caution. However, the drastic price drops for LIB technologies might indicate that a similar progression is possible for SSBs. In 2010, the average price for EV LIB technology was above 1200 €/kWh and has now fallen to prices as low as 90 €/kWh, while it is expected to fall below 45 €/kWh, a price drop equivalent to more than 96% [120,122]. SSBs can profit significantly from the price reductions for LIBs, as some components and production steps are closely related, especially for cathode manufacturing. Initially, SSBs will enter the market with decisively higher prices, originating from implementing new, initially more expensive materials with smaller or newly established value chains and different production methods [120].

**Figure 7:**
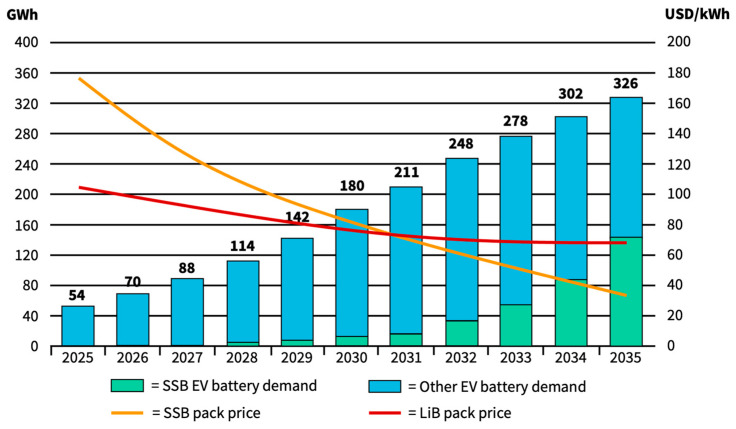
SSB and LIB battery demand and pack price forecast for 2025–2035 based on data available from [127,134]. USD was converted to EUR using a 1:1 ratio.

Figure 7 shows a cost progression forecast of SSB applications in EVs from Bloomberg NEF [127,134]. It is apparent that economic SSB progression is heavily dependent on economies of scale. This adds another factor of uncertainty to price forecasts since some components might be able to be taken over from conventional LIB technology. Still, mass production of SSBs is so far untested. Very little attention focuses on the challenges of processing air-sensitive glass/ceramic materials at giga-scale capacities with realistic geometries (thicknesses). Material cost, material performance, selection, and processing speed will all likely impact the ultimate application for SSBs [135]. However, according to Bloomberg NEF, economies of scale could lead to price parity between LIBs and SSBs as early as 2030, potentially inducing mass adoption of SSBs. Bloomberg NEF’s forecasted final SSB prices, approximately 35 €/kWh, may seem unrealistic from today’s perspective. Other authors, such as Hsieh et al., establish price targets for LIB packs using learning curves. These curves are influenced by cost parameters for the cathode production level and learning rates at the pack level [136]. Results are forecasts of 93 €/kWh at the lower end and 140 €/kWh at the higher end of the resulting cost range for 2030. Both values are higher than the cost curves presented in Figure 7 derived from Davidson et al. [134].

The final price of batteries is essentially based on their cost composition and the cost shares of specific processes along the value chain of SSB. As will be seen later, most projects are still in an (early) R&D phase. Indications about cost shares are, therefore, highly speculative. Accessible economic data is, in many cases, based on corporate statement releases, which are often unrepresentative, especially when considering the strong competition regarding SSB technologies. While an increase in energy density is a clear driver for the adoption of SSB in many markets, the commercial success of SSB will significantly depend on their material, processing, and production costs, which are often relatable to historic LIB economic parameters [120]. Generally, conventional battery manufacturing involves three primary processes: electrode production, cell production, and cell conditioning.

All these processes will be altered for SSBs and are highly dependent on the material properties of the solid electrolyte. The final product costs are mainly influenced by the cost of active and passive materials as well as cell manufacturing. Cell production and conditioning costs are mainly influenced by the choice of the SSE.

The total cost of cathode active materials will be comparable to liquid LIBs, since material costs are higher (e.g., 25 € kg^−1^ for LiNbO_3_), but coating layers are much thinner. In ASSBs, the cost of separators is around 1.5 € m^−2^. SSEs are expected to cost 5–10 € kg^−1^ if organic solvents are used. Inorganic SSE prices cannot be transferred as easily to SSB applications. Here, research institutions have used the methodology of considering metal values in SEs. Germanium-based Ses have proven to be unfeasible from an economic point of view. Ti, La, and zirconium-based oxide Ses are relatively cheap, ranging from 6 to 13 € kg^−1^. The metal cost approximation helps to estimate future costs by applying cost factors to them, ranging between 20 and 50% (depending on the electrode purity level required). The future costs of sulfide-based SSB technologies compared to oxide-based technologies cannot yet be forecasted well, since no supply chain of these materials currently exists. It can be seen from such forms of analysis that the metal value of the SE alone (3–6 € kWh^−1^) is already higher than the cost of the entire electrolyte in liquid LIB technologies (3–8 € kWh^−1^). To make SSBs cost competitive, it is therefore mandatory to bring SE’s final material costs as close to the final metal values as possible [120].

**Figure 8:**
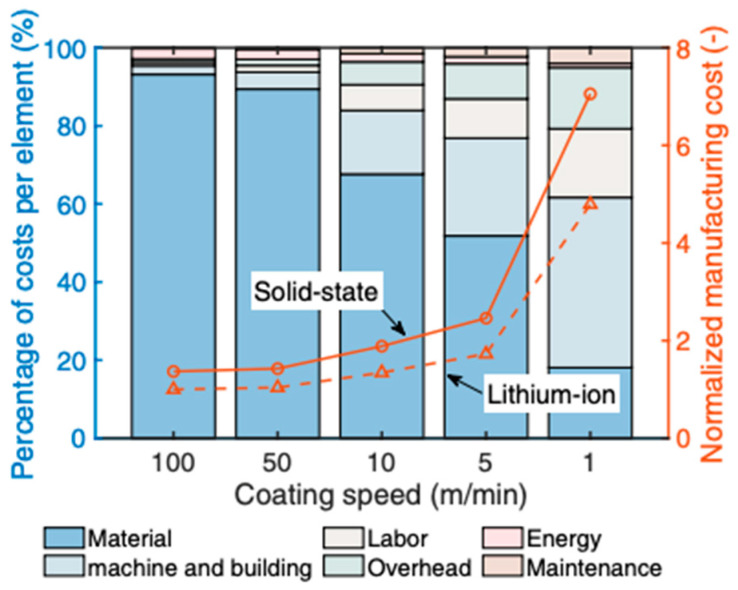
Cost breakdown of the battery manufacturing process based on electrode coating speed [135].

Furthermore, to achieve giga-capacities and SSB EV market penetrations, the electrode coating costs are essential since these are higher than conventional LIB in all process forms (but comparable). Hatzell and Zeng demonstrated that electrode manufacturing costs are always dependent on the coating speed, with higher coating speeds shifting the cost ratios heavily towards higher capital expenditures (CAPEX) costs (see Figure 8) [135]. Some costs can be saved in SSB cell manufacturing compared to liquid LIBs by reducing the number of process steps or times (for example, the reduction in formation and aging times).

On the other hand, there will most likely be new manufacturing steps for SSB for which innovative state-of-the-art manufacturing equipment must be developed. Especially during the ramp-up phase of SSB commercialization, this means higher investments in production infrastructure per GWh capacity since no standardized turnkey solutions exist today. However, in an up-scaled production as well, any additional processing steps can result in higher scrap rates as well as higher energy and material costs. High-temperature sintering steps for oxide SE materials are particularly critical [120].

As stated before, investments in the range of billions can be expected to build up production capacities globally. LIB production costs must be undercut to make SSB production economically feasible and justify large-scale investments in emerging technologies during times of high global inflation rates, as seen in Figure 7. Since new SSB factories will be CAPEX-intensive, production must run for time horizons of at least double-digit years to offset higher-than-before interest rates. Manufacturing synergies with existing LIB production could turn out to be crucial for lowering the final cost. In the future, SSBs will likely adopt manufacturing approaches from both the solid oxide fuel cell and conventional battery manufacturing community [120,135].

### 5.4. Key SSB Players and Collaborations

**Figure 9:**
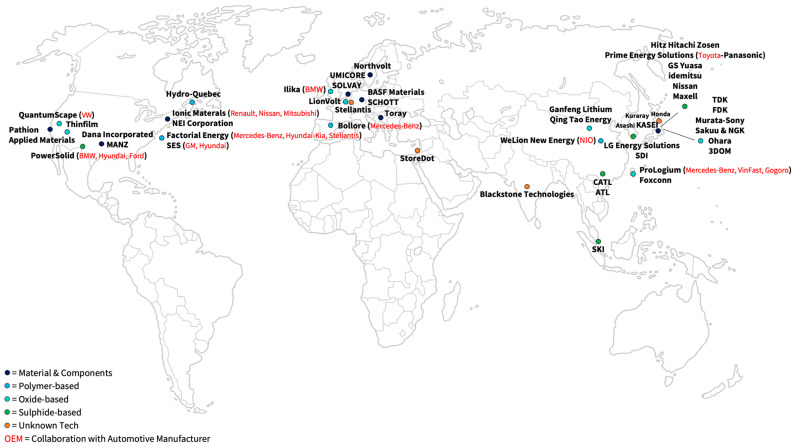
Overview of current key SSB market players regarding the technologies used.

The global SSB market is strongly dominated by R&D activities. Key players can generally be divided regarding their respective SSB technology type (e.g., polymer-/oxide-/sulfide-based). An overview of the key market players is presented in Figure 9. More in-depth descriptions of the most important companies and their current ambitions are provided in Table 3, Table 4 and Table 5.

## 6. Conclusions

SSBs are highly promising upcoming battery technologies. It is a novel technology vital in shaping the future of energy and sustainability. SSBs differ greatly from widely employed Li-ion batteries by using solid electrolytes instead of liquid ones due to their enhanced safety, higher energy density, and longer lifespans.

The development of solid electrolytes is crucial for SSBs and has advanced significantly, with inorganic and organic solid electrolytes each offering unique benefits but also facing notable limitations. Inorganic solid electrolytes such as LLZO excel in ionic conductivity and thermal stability but are less flexible and compatible with certain materials. Alternatively, organic solid electrolytes, such as PEO, are highly flexible and compatible but exhibit lower ionic conductivity and thermal stability. To optimize properties, CSEs that blend inorganic and organic components have emerged as a promising solution, harnessing the strengths of both while mitigating their weaknesses, thus ushering in a new era of safer, higher-energy-density SSBs.

Electrode materials for SSBs are also critical in determining their performance and safety. In the context of anodes, Li metal is a popular choice due to its high theoretical capacity, lightweight properties, and low electrochemical potential. However, it faces challenges such as reactivity, susceptibility to oxidation, and dendrite formation, which can pose safety concerns. Researchers are actively exploring alternative anode materials like Si, S, metallic alloys, and carbon-based substances to overcome these limitations and improve overall battery performance. On the other hand, cathode materials significantly impact energy density and overall battery performance. Materials like LiCoO_2_, LiFePO_4_, and LiNiMnCoO_2_ are commonly used due to their high energy densities, stability, and relative cost-effectiveness. The choice of cathode material is closely tied to the selected electrolyte, and researchers are exploring various strategies to optimize the connections between solid electrolytes and cathodes. Inorganic solid electrolytes have shown promise with nickel-rich cathodes in traditional LIBs, although density-related challenges hinder their application in SSBs. Innovative approaches, such as single-crystalline structures, aim to address these challenges.

The global battery market is forecast to grow exponentially, driven by EVs, and SSBs represent an emerging sector within this market. The current use of SSBs is limited, and market share is expected to be just over 1% by 2035, while LIBs are expected to dominate the market. Technological challenges, uncertainties, and high initial costs characterize the development of SSBs. Still, they promise higher energy density that addresses key consumer concerns such as range and charging times for electric vehicles. Achieving economies of scale is critical for SSBs to reach price parity with LIBs by 2030, which could lead to mass adoption. The production and commercial success of SSBs is highly dependent on reducing material, processing, and production costs, and innovations in manufacturing methods are crucial. Despite differing market and cost structure projections, there is a consensus on the significant growth in the SSB sector, supported by extensive patent activity and R&D investment. While the market penetration of SSBs depends on outperforming LIB production costs and significant investments in production infrastructure, synergies with existing LIB production and technological advances can potentially accelerate the commercialization and adoption of SSBs by leveraging their inherent advantages in energy storage solutions.

## Figures and Tables

**Table 1:** Ionic conductivity of typical inorganic and organic electrolytes.

Material	Ionic Conductivity */S cm^−1^ at RT	Source
LATP	10^−3^	[75]
LLZO	10^−6^–10^−3^	[51]
LTTO	10^−3^	[32]
LISICON	10^−6^–10^−4^	[51]
LPS	10^−2^	[47]
LGPS	10^−2^	[52]
PVDF	10^−8^–10^−6^	[68]
PEO	10^−8^–10^−6^	[68]
PAN	10^−8^–10^−6^	[68]

* The actual values of ionic conductivity can vary depending on various factors, such as the composition, processing conditions, and measurement methods [15].

**Table 2:** Ionic conductivity of typical inorganic filler-based composite solid electrolytes.

Compound	Ionic Conductivity/S cm^−1^ at T/°C	Source
SiO_2_/PPC/LiTFSI	8.5 × 10^−4^/60 °C	[82]
SiO_2_ NTs/PEO/LiTFSI	4.35 × 10^−4^/30 °C	[77]
SiO_2_ NFs/PEO-LiTFSI-SN	1.3 × 10^−4^/30 °C	[83]
LLZO/PEO/LiTFSI/PEGDME	4.7 × 10^−4^/60 °C	[84]
LLZO NWs/PEO/LiTFSI	2.39 × 10^−4^/RT	[85]
Li/LATP-3D/LiFePO_4_	7.47 × 10^−4^/60 °C	[80]
LLZAO-PEO/LiClO_4_	2.25 × 10^−5^/30 °C	[79]
LLTO/PEO	3.31 × 10^−4^/RT	[86]
LLTO/PAN-PVDF	1.43 × 10^−3/^RT	[87]

**Table 3:** Overview of the most active polymer-based SSB market players in R&D institutions and battery industries.

Player	Description	OEM Collaboration	Year of Market Entry	Source
Bollore (BlueSolutions)	Bollore developed a passenger car with an SSB (BlueCar) in 2011. In addition, buses equipped with SSBs were launched in 2020 together with Mercedes. The Bollore-owned company “BlueSolutions” has been selling LMP technology (all-solid-state Li-metal polymer) since 2011. It is now selling its SSBs to Daimler for the eCitaro-bus.	Mercedes-Benz	2020	[119,120]
WeLion New Energy Technology	Car manufacturer NIO, together with WeLion New Energy Technology, launched a polymer battery with a Li metal anode and an NMC cathode in 2022. It also announced the start of construction of a production plant that will initially produce 20 GWh of hybrid SSBs with liquid electrolyte, as well as ASSB. An expansion to 100 GWh is targeted.	NIO	2022	[119,120]
Factorial Energy	Factorial Energy presented a cell with a solid separator, liquid electrolyte, and a Li metal anode that achieved 40 Ah capacity in 2021. The OEM Hyundai-Kia, Mercedes-Benz, and Stellantis have already invested in Factorial Energy. Mercedes-Benz and Factorial Energy scheduled a small series to enter the market for automotive applications by the end of 2026.	Hyundai-Kia, Mercedes-Benz, Stellantis	2026	[119,120]
Solid Energy Systems	Solid Energy Systems plans to develop a prototype car together with GM and Hyundai by 2023 and reach market maturity by 2030. SES relies on a hybrid cell concept with an LCO or an NCA cathode active material and a Li metal anode.	GM, Hyundai	2030	[119,120]
Hydro-Quebec	Hydro Quebec plans to start production between 2025 and 2027, initially launching polymer electrolytes with a Li metal anode and an LFP cathode. Later, the LFP cathode will be replaced with NMC, and the polymer electrolyte will be replaced by a composite electrolyte with ceramic components.	Mercedes-Benz	2025–2027	[119,120]
Ionic Materials	Furthermore, the company Ionic Materials is developing a polymer battery with Renault-Nissan-Mitsubishi. Ionic Materials is known to only use a Li metal anode. A123 Systems LLC will invest in the project.	Renault, Nissan, Mitsubishi	-	[119,120]

**Table 4:** Overview of the most active oxide-based SSB market players in R&D institutions and battery industries.

Player *	Description	OEM Collaboration	Year of Market Entry	Source
QuantumScape	In another cooperation between carmaker VW and cell manufacturer Quantum Scape, market-ready batteries for the automotive sector will be developed by 2025. In 2024, Quantum Scape will build up a production capacity of 1 GWh, which will be expanded to 20 GWh by 2026. QuantumScape describes the electrolyte material as ceramic and has already demonstrated prototype cells with Li anodes. Due to the potential proximity to oxide materials, the announcements are classified as oxides.	VW	2024	[119,120]
ProLogium	Cell maker ProLogium and car manufacturers are teaming up to put an SSB in a commercial vehicle (VinFast) or Prototypes (Mercedes-Benz) by 2023. For this goal, production capacities of 1 to 2 GWh were planned to be built up in 2022. A battery with a ceramic separator and a capacity of 2.5 kWh was demonstrated together with scooter manufacturer Gogoro in 2022.	VinFast, Mercedes-Benz, Gogoro	2023	[119,120]
Ganfeng Lithium	Ganfeng Lithium is one of China’s largest battery producers. The Li and battery manufacturer started to build a 10 GWh SSB factory in 2022 with a second 10 GWh factory planned to produce SSBs with 360 Wh/kg.	-	-	[137]
Qing Tao Energy	Qing Tao Energy Development and Ampcera are also working on solid oxide electrolytes. Quin Tao announced a production capacity of 1 GWh in 2020 and a second production facility with an optional capacity of 10 GWh in 2022.	SAIC Motor	-	[119,120]
Ilika	Founded in 2004, Ilika started designing the Stereax family of mm-scale SSBs for medical implants and industrial IoT devices in 2014. Financed by three rounds of venture capital, the company was publicly listed in 2010. The company currently plans to start its MWh scale-up of EV batteries.	BMW	-	[119,120,138]

* Other notable players include STMicroelectronics, LionVolt, Murata-Sony, TDK, FDK, Sakuu & NGK, Ohara, 3DOM, and Foxconn [119,120].

**Table 5:** Overview of the most active sulfide-based SSB market players in R&D institutions and battery industries.

Player *	Description	OEM Collaboration	Year of Market Entry	Source
Samsung SDI	In 2020, SDI introduced a prototype cell with an in situ Li-metal anode and started the construction of a pilot production plant in 2022.	-	2027	[119,120]
CATL	According to the company‘s own roadmap, CATL plans to be the first SSB cell manufacturer by developing a sulfide SSB ready for market introduction by 2025.	-	2025	[119,120]
LGES	SKI has announced their SSB will be ready for market penetration by 2030.	-	2030+	[119,120]
PowerSolid	In addition, PowerSolid plans to develop a prototype car with an SSB before 2025 and a series-produced SSB for passenger cars by the end of the decade in collaboration with BMW and Ford. Since 2018, Hyundai has also taken a financial stake in PowerSolid. PowerSolid plans to develop a 100 Ah cell with a Si anode by 2026 and a 100 Ah cell with a Li metal anode by 2028, with investments from A123 Systems LLC.	BMW, Ford, Hyundai	2025	[119,120]
Prime Planet Energy	The cooperation between Toyota and Panasonic presented a prototype of a car equipped with an SSB in 2021. Although no technical data were published on this battery, it can be assumed that a sulfide electrolyte was used. They plan to bring the SSB to the market by 2025.	Toyota, Panasonic	2025	[119,120]

* Other notable players include Hitz Hitachi Zosen, Idemitsu, ATL, LG Energy Solutions, GS Yuasa, Nissan (own development), Honda (own development), and Maxell [119,120].

## Data Availability

The data presented in this study are available on request from the corresponding author.
